# Population genetic structure and conservation management of hill pigeons (*Columba rupestris*) recently endangered in South Korea

**DOI:** 10.1007/s13258-021-01212-x

**Published:** 2022-01-13

**Authors:** Jin-Yong Kim, Soo Hyung Eo, Seung-Gu Kang, Jung Eun Hwang, Yonggu Yeo, Jongmin Yoon

**Affiliations:** 1 Research Center for Endangered Species, National Institute of Ecology, Yeongyang, South Korea; 2 Department of Forest Science, Kongju National University, Yesan, South Korea; 3 Conservation and Health Center, Seoul Zoo, Gwacheon, South Korea

**Keywords:** Conservation, Domestic pigeon, Hill pigeon, Hybridization, mtDNA, Population genetics

## Abstract

**Background:**

Hill pigeons (*Columba rupestris*) are close to local extinction (ca. less than 100 individuals) in South Korea where a variety of conservation management procedures are urgently required.

**Objective:**

This study was aimed at determining the conservation direction of captive propagation and reintroduction of hill pigeons using genetic information based on mitochondrial DNA. We also evaluated the extent of hybridization between hill pigeons and cohabiting domestic pigeons.

**Methods:**

We used 51 blood samples of hill pigeons from Goheung (GH), Gurye (GR), and Uiryeong (UR), and domestic pigeons cohabiting with hill pigeon populations. Genetic diversity, pairwise *Fst*, analysis of molecular variance, and haplotype network analysis were used to examine the genetic structure of hill pigeons.

**Results:**

Hill pigeons that inhabited South Korea were not genetically distinct from Mongolian and Russian populations and showed relatively low genetic diversity compared with other endangered species in Columbidae. The GR population that exhibited the largest population size showed lower genetic diversity, compared to the other populations, although the pairwise *Fst* values of the three populations indicated low genetic differentiation. The GH and GR populations were confirmed to lack hybridization, *relatively*, whereas the UR population was found to exhibit some degrees of hybridization.

**Conclusion:**

To conserve hill pigeons with low genetic diversity and differentiation in South Korea, the conservation process of captive propagation and reintroduction may require artificial gene flows among genetically verified populations in captivity and wildness. The introduction of foreign individuals from surrounding countries is also considered an alternative strategy for maintaining genetic diversity.

**Supplementary Information:**

The online version contains supplementary material available at 10.1007/s13258-021-01212-x.

## Introduction

Endangered birds are threatened, and their locally fragmented populations have been decreasing due to numerous factors, including habitat degradation, overexploitation, negative interspecific interaction, and climate change, which have affected their survival and reproduction (Brook et al. 2008; Steven and Castley 2013). A decrease in population size may lead to a reduction in the degree of genetic diversity, and the extent of this reduction in the rate of population growth depends on the severity of bottleneck events (Nei et al. 1975; Heber and Briskie 2010). The negative fitness consequences of reduced genetic diversity and/or inbreeding depression can deteriorate fertility and embryonic development (Keller and Waller 2002; Heber and Briskie 2010), and eventually accelerate species extinction. Some threatened species are also affected by hybridization with cohabiting closely related species, presumably resulting in fitness reduction (Wolf et al. 2001; Haig et al. 2004) or an increase in polymorphisms, which allow rapid evolution with some fitness-related benefits (Grant and Grant 1992; Rhymer et al. 1994; Haig et al. 2004).

The hill pigeon (*Columba rupestris*) has an extremely large range (southern Russia, southern China, Mongolia, southern Tibet, and the Korean Peninsula) (Baptist et al. 2020) and is globally evaluated as under “Least Concern” by IUCN (International Union for Conservation of Nature). However, the population size of hill pigeons continuously decreased in South Korea after the late twentieth century, and the species were hence designated as “Endangered Species Grade II” by the Korean Ministry of Environment in 2017. Previous studies revealed that the total population size of hill pigeons is as small as ca. 100 individuals in locally fragmented regions (Ministry of Environment, 2016). Therefore, hill pigeons in South Korea are considered to have limited breeding opportunities, and genetic problems such as genetic drift and/or inbreeding depression caused by a decrease in the level of genetic diversity in the gene pool. Moreover, hybridization between hill pigeons and domestic pigeons (*C. livia domestica*), which are a closely related species have been found while small numbers inhabited with hill pigeons, resulting in reproduction and resource competition (Soares et al. 2016). Specifically, it has been more frequently identified in Uiryeong County than in Gurye and Goheung Counties of South Korea, and the frequency of hybridization appeared to increase over the populations. The identification of hybrids is the first step in evaluating the extent of hybridization (Haig et al. 2004). There is currently a lack of understanding of the genetic characteristics as well as ongoing hybridization of hill pigeons, so the related ecological issues need to be discussed.

For the long-term conservation of endangered species, the maintenance of genetic diversity and the determination of meaningful management based on genetic structure are essential (Ando et al. 2014). The habitats of hill pigeons are separated into only three regions (Gurye, Goheung, and Uiryeong) in South Korea (Ministry of Environment, 2016). Determining whether or not a population has a separate evolutionarily significant unit is an important criterion for planning *ex-situ* conservation strategies including captive propagation and reintroduction of hill pigeons. Therefore, the aspect of genetic differentiation among regions plays an important role in decision-making for the reintroduction of endangered species. Information on a wider range of genetic differentiation (e.g., among South Korean, Russian, and Mongolian populations) should also be provided for the reintroduction of species and captive propagation.

This study was aimed at analyzing the conservation management of the hill pigeon populations endangered in South Korea using data on genetic structure based on mitochondrial DNA (mtDNA). To this end, we verified (1) the genetic diversity in the three wild populations (Goheung, Gurye, and Uiryeong regions) of South Korea, (2) the genetic distance and differentiation among the three global populations (Mongolia, Russia, and South Korea), and (3) the haplotype network among the three populations of hill pigeons and cohabiting domestic pigeons to evaluate the extent of hybridization.

## Material and methods

### Sampling and DNA extraction

**Fig. 1:**
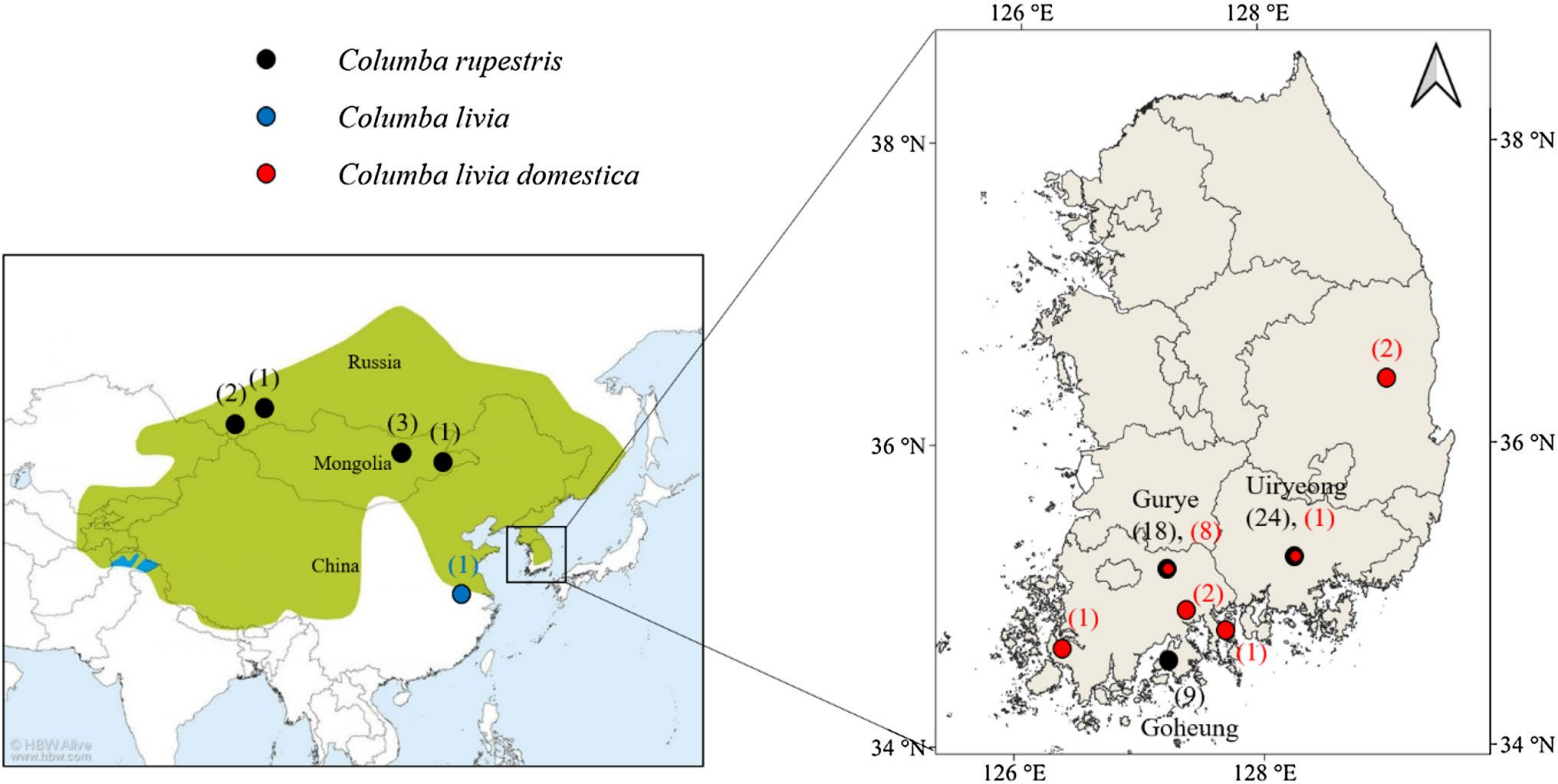
Global distribution of the Hill pigeon (*C. rupestris*) based on data collected from the IUCN (https://iucnredlist.org) and sampling sites; values in parentheses indicate the numbers of samples

Sampling of wild populations was carried out at Goheung, Gurye, and Uiryeong in South Korea between 2019 and 2021 (Fig. 1). The captured individuals were transferred to the Research Center for Endangered Species, South Korea for captive propagation after blood extraction and ring attachment, or released back to the site. We collected 51 blood samples of *C. rupestris*, from Goheung (GH, n = 9), Gurye (GR, n = 18), and Uiryeong (UR, n = 24), and *C. livia domestica* (LD, n = 15) from the three wild populations. The collected samples were stored at -30 °C before DNA extraction. DNA was extracted using the DNeasy Blood & Tissue Kit (Qiagen, Hilden, Germany) followed by the manufacturer’s protocol. Cytochrome oxidase subunit 1 (COI), cytochrome b (cyt b), and D-loop sequences of the Hill pigeon and Rock pigeon were downloaded from the NCBI database (https://www.ncbi.nlm.nih.gov/) (Table 1).

**Table 1:** Profiles of samples collected from the wild populations of hill pigeons (*Columba rupestris*) and domestic pigeons (*C. livia domestica*) in South Korea and also downloaded from NCBI

Species	mtDNA Region	Population	No. of samples	Sources; collecting locality	Accession No
*Columba rupestris*	COI	Russia	1	Ovyurskiy Kozhuun, Tuva, Russia	GQ481614.1
			2	Mongun-Taiginskiy Kozhuun, Tuva, Russia	GQ481612.1 GQ481610.1
		Mongolia	3	Bayan Ovoo, Hentiy, Mongolia	NC_031867.1 (reference sequence)
					GQ481613.1
					GQ481615.1
			1	Dornod Aymag, Dornod, Mongolia	GQ481611.1
		South Korea	47	Goheung, Gurye, and Uiryeong, South Korea	In this study
	Cyt b and D-loop	Goheung	9	Goheung, Jeollanamdo, South Korea	In this study
		Gurye	18	Gurye, Jeollanamdo, South Korea	In this study
		Uiryeong	24	Uiryeong, Kyungsangnamdo, South Korea	In this study
		Mongolia	1	Bayan Ovoo, Hentiy, Mongolia	NC_031867.1 (reference sequence)
*Columba livia domestica*	Cyt b and D-loop	South Korea	15	Gurye, Uiryeong, Yeongyang, Mokpo, Guangyang, and Yeosu, South Korea	In this study
*Columba livia*	Cyt b and D-loop	China	1	Wuhu, Anhui, China	NC_013978.1 (reference sequence)

### PCR amplification and sequencing

The target mitochondrial genes consisted of DNA sequences from the cyt b and D-loop regions, which have been shown to provide sufficient sequence variation to resolve population structure in pigeons and other birds (Johnson and Clayton 2000; Goldberg et al. 2011). We amplified a 1272 bp fragment of the D-loop region using the primers KXH0, KXH5, Av438FDloopB, Av1301R12S, 129_pigeonF, and 317_pigeonR (Table 2). We excluded approximately 492 bp adjacent to the 12S rRNA gene because of tandem repeats (Goldberg et al. 2011). The primers Hill_DloopF and Hill_DloopR were redesigned to optimize the amplification of the *C. rupestris* D-loop region using the primers Av438FdloopB and Av1301R12S (Table 2). We also sequenced a 988 bp cyt b DNA region. Primers for the cyt b amplification were designed using the reference genome sequence of *C. rupestris* mtDNA (GeneBank accession number: NC_031867) and Geneious Prime (Biomatters, Auckland, New Zealand). Although COI has generally been chosen as the standard barcoding marker due to its high interspecific variation, it can also provide clues about ongoing speciation processes and direct attention to species or species complexes (Johnsen et al. 2010). We amplified a 603 bp fragment of the COI region using the primers Bird F1 and Bird R1 (Johnsen et al. 2010). PCR amplifications for all the samples were carried out with 30 µL volumes, using 2 × Lamp Taq PCR Pre-Mix (Biofact, Daejeon, Korea). The PCR thermal profile for the COI, cyt b, and D-loop sequences started with 94 °C for 4 min followed by 35 cycles of 94 °C for 1 min, 58 °C for 1 min, and 72 °C for 1 min for all the reactions. All the PCR products were checked on 1% agarose gels. The sequencing was carried out by a commercial sequencing service (Macrogen Inc., Seoul, South Korea).

**Table 2:** Basic information of primers used in the genetic analyses for hill pigeons and domestic pigeons

Region	Name	Sequences (5′ – > 3′)	References
COI	BirdF1 BirdR1	TTCTCCAACCACAAAGACATTGGCAC ACGTGGGAGATAATTCCAAATCCTG	Johnsen et al. (2010)
Cyt b	Hill_cytbF	TTCTATCCCTCCATAGACCTGT	In this study
	Hill_cytbR	CGATGTTTTCATAAACTATTAGAGT	
D-loop	KXH0	TGTCCTATGTACTACAGTGCATCG	Ando et al. (2014)
	KXH5	ATGGCCCTGACATAGGAACCAGAG	Ando et al. (2014)
	Hill_DloopF	TCACGTGAAACCAGCAACTC	Goldberg et al. (2011)
	Hill_DloopR	GGTAAGGTTAGGA CTAAGTC	Goldberg et al. (2011)
	129_pigeonF	CCATTTTAGTCCGTGATCGC	Goldberg et al. (2011)
	317_pigeonR	AGTGCA TCAGTGTAAAGGTG	Goldberg et al. (2011)

### Data analyses

All analyses were based on concatenated cyt b and D-loop sequences (South Korea, n = 51), except that of pairwise *Fst* information from Mongolia (n = 4), Russia (n = 3), and South Korea (n = 47), which was based on COI sequences. Haplotype diversity (*h*), nucleotide diversity (*π*), the number of polymorphic sites (*S*), and the average number of nucleotide differences (*k*) were calculated using DNA SP V.6.12.03. The population pairwise genetic distance *Fst* values and their significance were calculated in Arlequin 3.5.2. using 1,000 permutations of haplotypes among populations used to construct a null distribution for significance testing. Heat maps of the population pairwise *Fst* values were generated using R statistical software v4.1.0. An analysis of molecular variance (AMOVA) was implemented in Arlequin 3.5.2. to test whether a significant proportion of genetic variation was present. Haplotype network analysis was conducted in PopART 1.7 using a median-joining network.

## Results

### Genetic diversity of wild populations

The haplotype and nucleotide diversities of three populations in South Korea were calculated (Table 3). The haplotype diversity of GH was the highest (*h* = 0.917, Table 3). However, the number of individuals of GH was lower than those of the other populations; nine samples belonged to seven different haplotypes. The *h* of GR was the lowest (*h* = 0.752, Table 3). The average number of nucleotide differences and nucleotide diversity were the highest in UR (*k* = 7.500, π = 0.00332; Table 3). GR showed the lowest values of *k* and *π* (*k* = 2.346, π = 0.00104; Table 3). *π* was more than three times higher for UR, compared with GR. The *h* value of the total population (GH, GR, and UR) in South Korea was 0.874, and the *π* value was 0.00226 (Table 3).

**Table 3:** Summary statistics of molecular diversity for three populations (GH, GR, and UR) of hill pigeons in South Korea, based on concatenated cyt b and D-loop data

Population	n	*N*_hap_	*bp*	*k*	*S*	*h*	*π*
GH	9	7	2260	3.833	13	0.917	0.00170
GR	18	8	2260	2.346	11	0.752	0.00104
UR	24	11	2260	7.500	43	0.862	0.00332
GH + GR + UR	51	20	2260	5.104	55	0.874	0.00226

*n* number of individuals, *N*_hap_ number of haplotypes, *bp* base pair, *k* average number of nucleotide differences, *S* number of polymorphic sites, *h* haplotype diversity, π nucleotide diversity

### Genetic distance and differentiation among wild populations

**Fig. 2:**
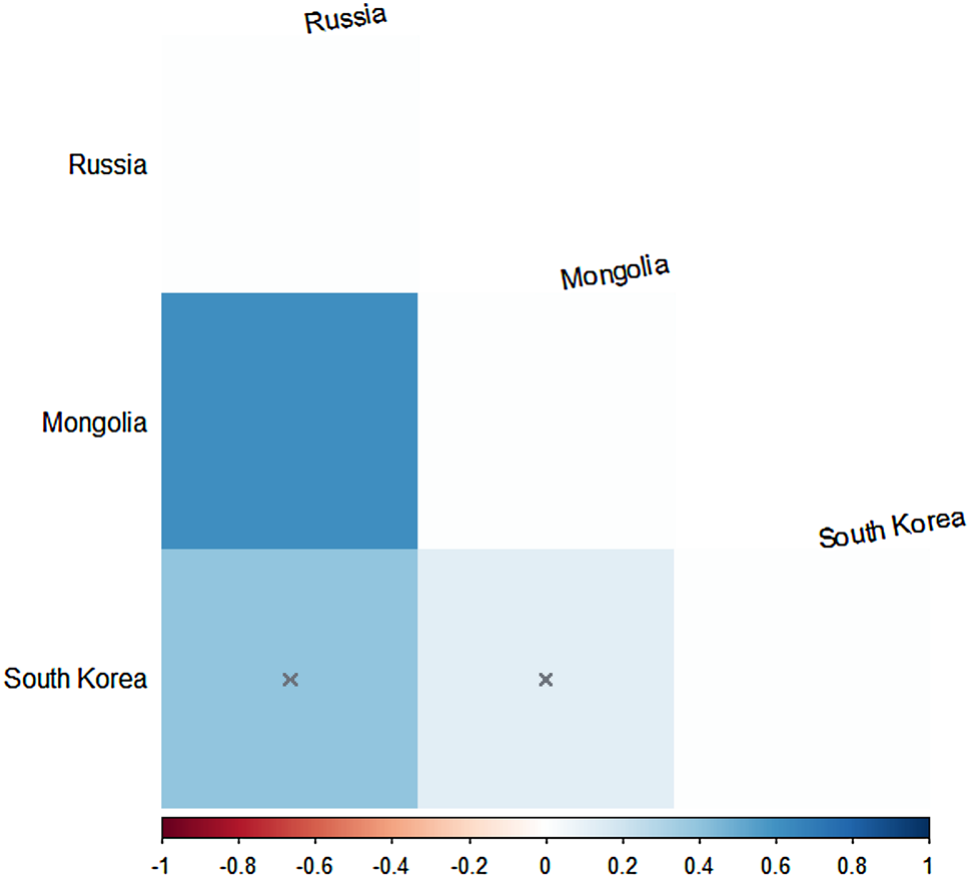
Heat map of pairwise *Fst* values for three populations of *C. rupestris* based on COI data; highlighted values (x) indicate non-significance

**Fig. 3:**
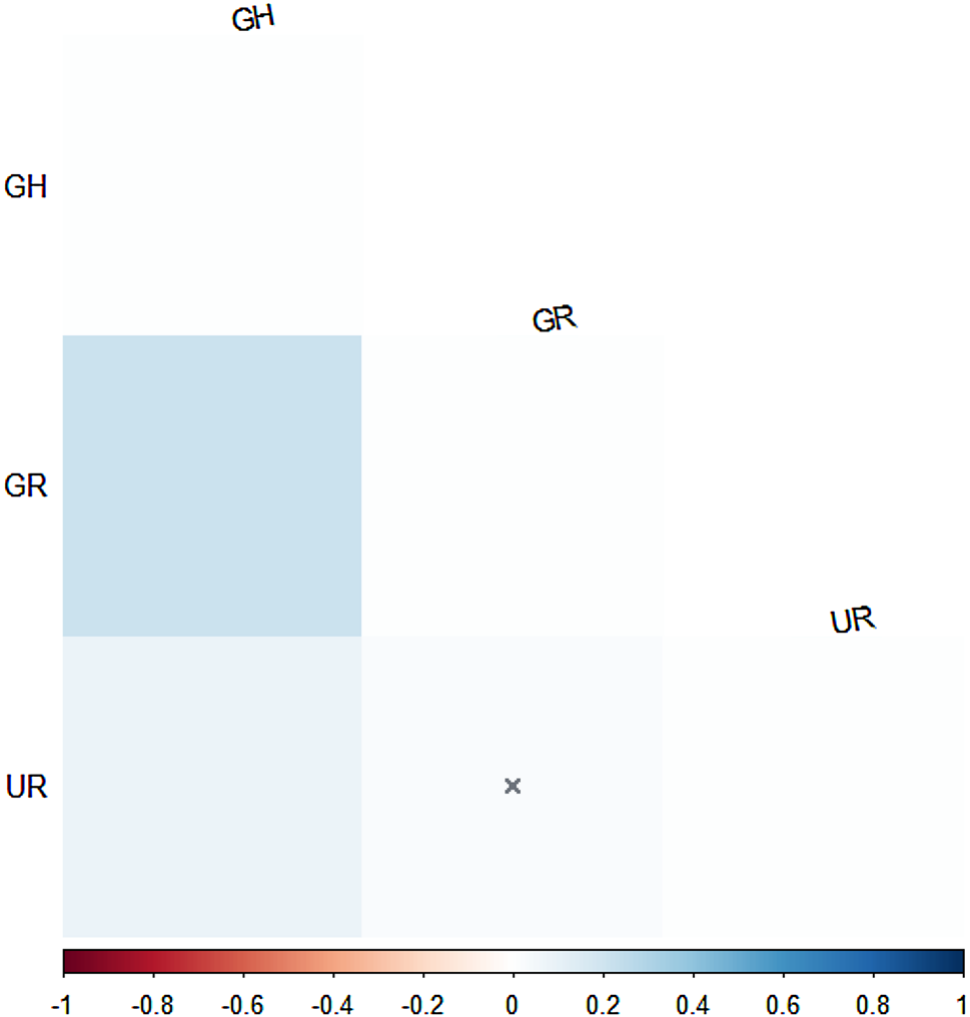
Heat map of pairwise *Fst* values for three populations (GR, GH, and UR) of *C. rupestris* in South Korea based on concatenated cyt b and D-loop data; highlighted values (x) indicate non-significance

First, prior to analyzing the genetic distance, the phylogenetic tree was verified using the closely related species *C. guinea, C. plumbus, Stereptopelia chinensis*, and *S. orientalis* (Johnson et al. 2001) (Supplementary Fig. 1). Our results of pairwise *Fst* based on the COI gene (Fig. 2) showed that the South Korean population was not genetically distinct from the Mongolian population (*Fst* = 0.120, *P* = 0.099); the pairwise *Fst* value of the South Korean and Russian populations was 0.400, but this was non-significant (*P* = 0.261). The Russian population was genetically distinct from the Mongolian population (*Fst* = 0.613, *P* = 0.036). Second, the pairwise *Fst* values of the three populations of hill pigeons in South Korea (Fig. 3) showed low genetic differentiation. Although the pairwise distance between the GH and GR populations showed a significant relationship, the *Fst* value showed low genetic differentiation (*Fst* = 0.21). The UR population was not significantly different from the GR population (*Fst* = 0.02); these populations showed a significant relationship but low genetic differentiation (*Fst* = 0.08). Overall, the results of AMOVA (Table 4) revealed small population variations, which accounted for only 6.88% of the total molecular variance. The within-population variation was 93.12% of the total molecular variance.

**Table 4:** AMOVA results for *C. rupestris* concatenated cyt b and D-loop regions for three wild populations (GH, GR, and UR) in South Korea

Source of variation	df	Sum of squares	Variance component	% variation	*P*-value	Fixation indices
Cyt b & D-loop						
Among populations	2	10.774	0.18314	6.88	0.02933	0.06881
Within populations	48	118.960	2.47833	93.12		
Total	51	139.734	2.66147			

### Haplotype network among wild populations

**Fig. 4:**
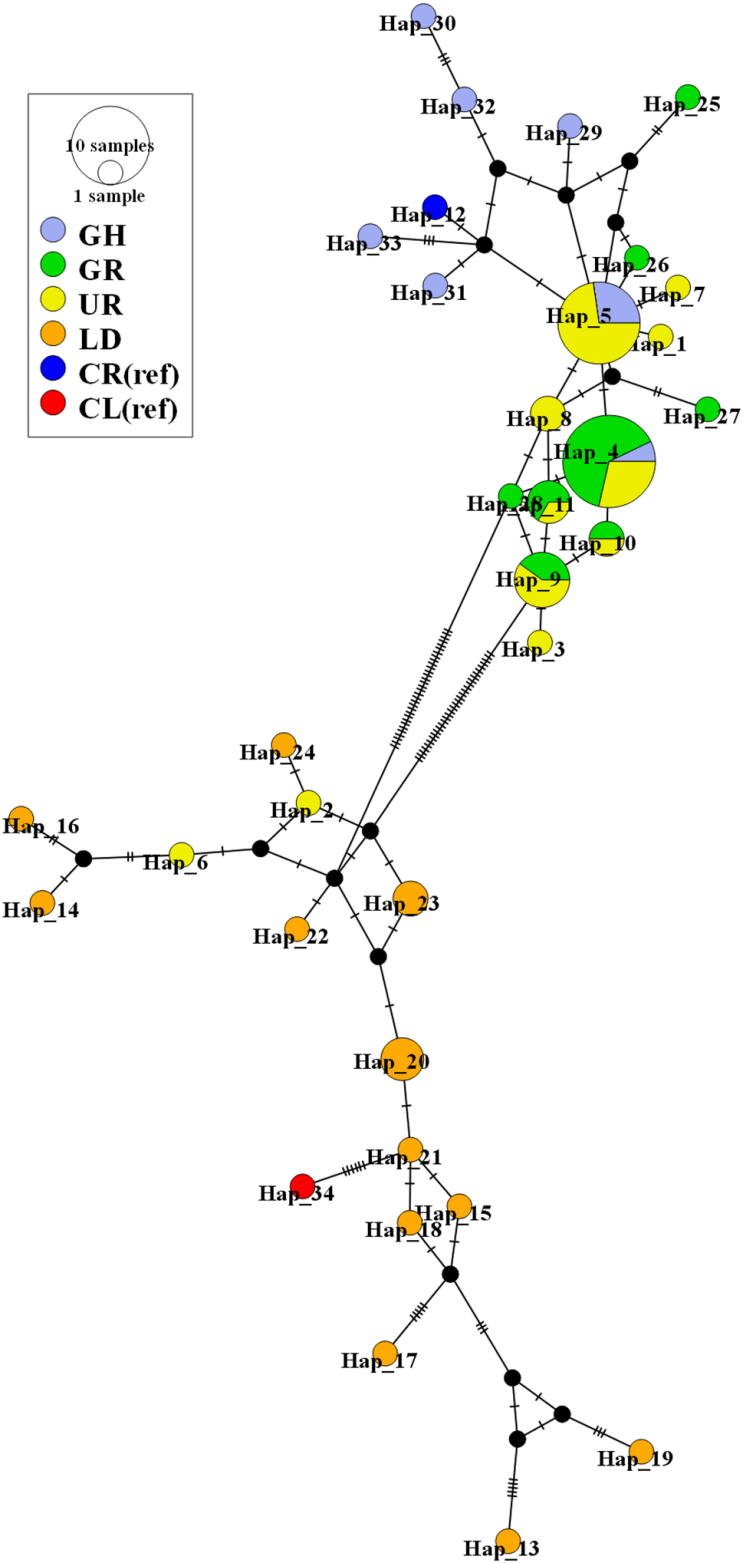
Median-joining network for *C. rupestris* and *C. livia domestica* haplotypes based on concatenated cyt b and D-loop data; a branch represents a single nucleotide change, and black dots on branches represent inferred missing haplotypes

To evaluate the extent of hybridization in South Korea populations, haplotype relationships were visualized using wild populations (*C. rupestris* and *C. livia domestica*) (Fig. 4). *C. livia* (reference sequence) that is closely related to *C. rupestris* (reference sequence), formed exclusive clades. All GH and GR individuals appeared in the *C. rupestris* clade, whereas all LD individuals appeared in the *C. livia* clade. Most UR individuals appeared in the *C. rupestris* clade, but 2 individuals labeled as Hap_2 and Hap_6 appeared in the *C. livia* clade. GR and UR had 4 identical haplotypes, and Haplotype_4 was identified as an identical type possessed by South Korea populations.

## Discussion

We verified the genetic diversity of hill pigeon populations locally endangered in South Korea using mitochondrial data. Our results of haplotype diversity in South Korea (*h* = 0.874; Table 3) do not seem to indicate a low haplotype diversity with respect to the available data from other endangered species of the family Columbidae. The lowest record was that of the red-headed wood pigeon (*C. janthina nitens*) (*h* = 0.12, π = 0.00105; Ando et al. 2014); the Puerto Rican Plain pigeon (*Patagioenas inornata*) showed an *h* value of 0.48 (Young and Allard 1997), and the smallest populations of the Japanese wood pigeon (*C. janthina*) showed an *h* range of 0.10–0.53 and a *π* range of 0.0009–0.0023 (Seki et al. 2007). However, our results of nucleotide diversity of South Korean populations indicated lower genetic diversity (π = 0.00226; Table 3) with respect to other endangered species of the Columbidae family. Among the South Korean populations, GR had the lowest genetic diversity (*h* = 0.752, π = 0.00104; Table 3), whereas UR had the highest genetic diversity (*h* = 0.862, π = 0.00332; Table 3). The GR population is currently known to be composed of relatively more wild individuals (ca. 50) compared with other populations. Small isolated populations may face an increasing risk of local extinction potentially due to the loss of genetic diversity (Vucetich and Waite 2000). The region for the GR population is expected to offer a suitable habitat for colonially breeding pigeons, but their low genetic diversity may pose a threat of inbreeding depression. Therefore, the GR population may need to increase its size through genetic diversity enhancement.

Hill pigeons with small numbers exhibited relatively low degrees of genetic distance and differentiation among the three populations in South Korea, but seemed to be genetically close to the Mongolian and Russian populations. For the genetic management of conserved or isolated populations, One-Migrant-per-Generation (OMPG) rule was suggested to offset genetic deterioration (Wright 1931; Slatkin 1987). The OMPG rule implies that the appropriate level of gene flow for maintaining genetic diversity and preventing inbreeding depression in fragmented populations is one migrant individual per local population per generation (Wright 1931; Mills and Allendorf 1996; Wang 2004). Recently, this rule was tested for real populations and found to be valid in experiments involving various taxa (Wang 2004). In the United States, for example, nearly every recovery plan that considers genetic issues and fragmentation applies the rule (Mills and Allendorf 1996; Wang 2004). It gives a specific number of migrants for populations, but one of its prerequisites is that small amounts of gene flow between partially isolated regions are necessary for long term persistence (Mills and Allendorf 1996). Our results of pairwise *Fst* showed that the South Korean populations were not genetically distinct from the Mongolian and Russian populations (Fig. 2). The *Fst* value among the Korean populations showed low genetic differentiation (Fig. 3), and that the GR population may be facing an increasing risk of inbreeding depression due to the ongoing loss of genetic diversity. According to our results for hill pigeons, an increase in the number of wild and captive individuals with the enhancement of genetic diversity through a small amount of gene flows among three populations in South Korea may be more beneficial than a conventional plan that preserves the genetic characteristics of the three locally fragmented populations. In addition, the South Korean populations did not show significant genetic differences compared with the Russian and Mongolian populations; some individuals from Mongolia and/or Russia would be helpful to reinforce captive propagation and reintroduction effort through artificial gene flow for the long-term conservation of hill pigeons in South Korea.

**Fig. 5:**
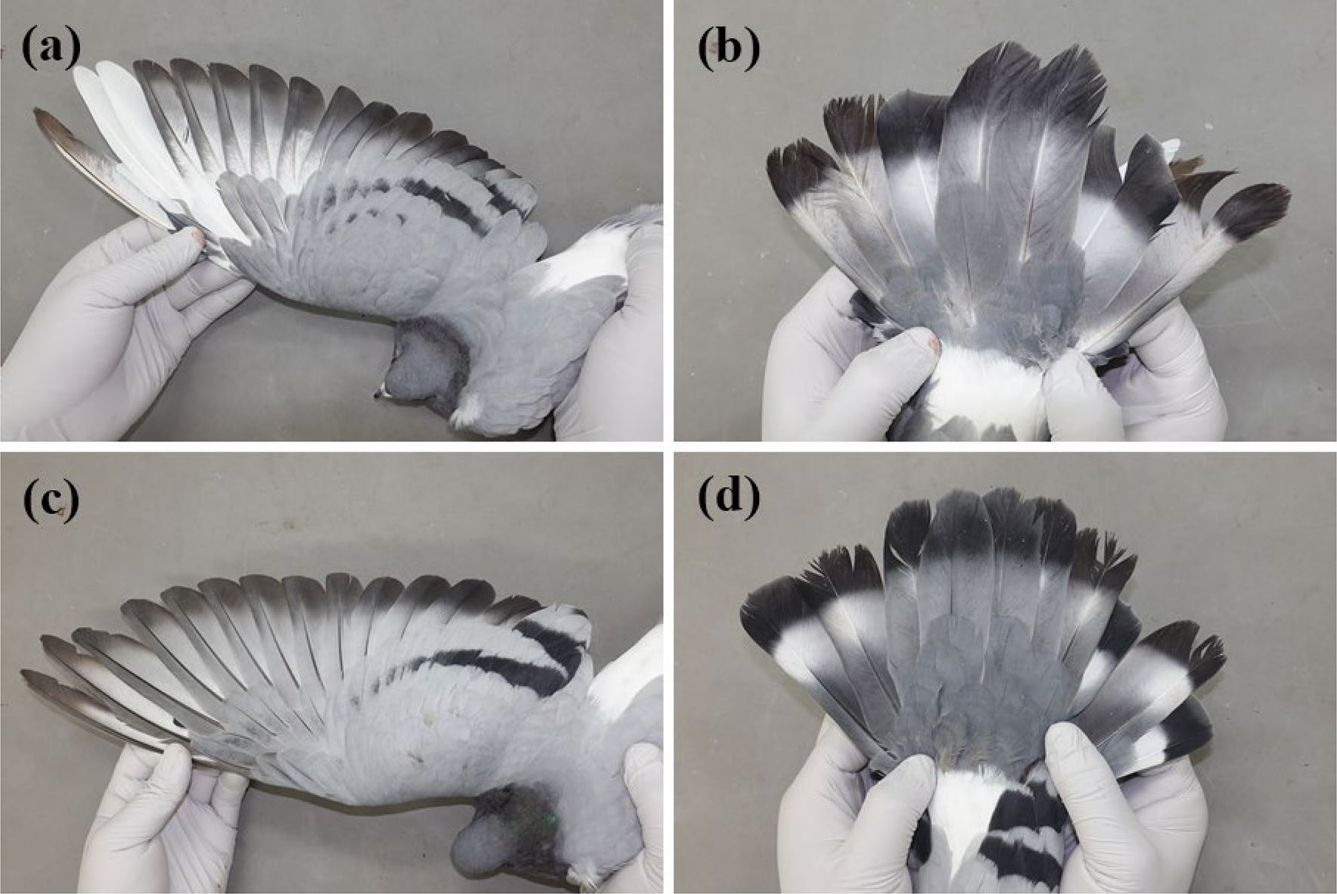
Phenotypes of suspected hybrids in UR population: wing (**a**) and tail (**b**) feathers of Hap_6 individual; wing (**c**) and tail (**d**) feathers of Hap_2 individual

Our results of haplotype network confirmed that the GH and GR populations were purebred hill pigeons, implying a lack of hybridization with domestic pigeons. However, it was suspected that the UR population was hybridized because two haplotypes of this population were included in the clade of a closely related species, the rock pigeon (*C. livia*) (Fig. 4). Thus, we confirmed the phenotype differentiation of two individuals belonging to these two haplotypes (Hap_2 and Hap_6). The observed phenotypes also showed a partial hybrid form (Fig. 5). Compared with those of purebred hill pigeons, the white line on the tail was not clear, and the black color of the wings was partially changed to white. We confirmed that some hybrid individuals could be sorted by utilizing mtDNA, but this was not a suitable marker for definitive sorting of hybrid individuals because of the maternal inheritance feature. Our results confirmed the hybridization of the UR population at the population level using mtDNA; however, more accurate marker development should be continued at the individual level using nuclear DNA in future studies.

## Conclusion

To suggest a conservation management strategy for the endangered hill pigeon populations in South Korea, we verified their genetic structure information using mtDNA. The nucleotide diversity of the South Korean populations indicated lower genetic diversity with respect to other endangered species of the Columbidae family. The GR population may be facing an increasing risk of inbreeding depression due to low genetic diversity. Our results of pairwise *Fst* showed that the South Korean populations were not genetically distinct from the Mongolian and Russian populations and the *Fst* value of the Korean populations showed low genetic differentiation. To increase the number of individuals, considering the enhancement in genetic diversity, a small amount of gene flow among the three populations in South Korea is required. In addition, introduction of Mongolian and Russian populations to make a new population in South Korea will also be a helpful conservation plan to increase the hill pigeon populations. The GH and GR populations were confirmed to be purebred hill pigeons, but the UR population was hybridized. We confirmed the hybridization of the UR population at the population level using mtDNA, but more accurate marker development using nuclear DNA is required.

## Supplementary Information

Below is the link to the electronic supplementary material.

Supplementary file1 (DOCX 46 KB)
