# Supplementary material for: Population genetic structure and conservation management of hill pigeons (Columba rupestris) recently endangered in South Korea
Source: Genes Genomics. 2022 Jan 13;44(11):1437–44. doi: 10.1007/s13258-021-01212-x (PMC9569314; doi:10.1007/s13258-021-01212-x)

**Supplementary Figure** 1. Phylogenetic tree of *Columba rupestris* populations, *Columba livia domestica* and three Columbidae bird as an out group. Numbers at nodes represent the bootstrap values for maximum likelihood analysis using COI. Unlabeled nodes received <10% bootstrap support. Branch lengths are proportional to likelihood estimated branch lengths.


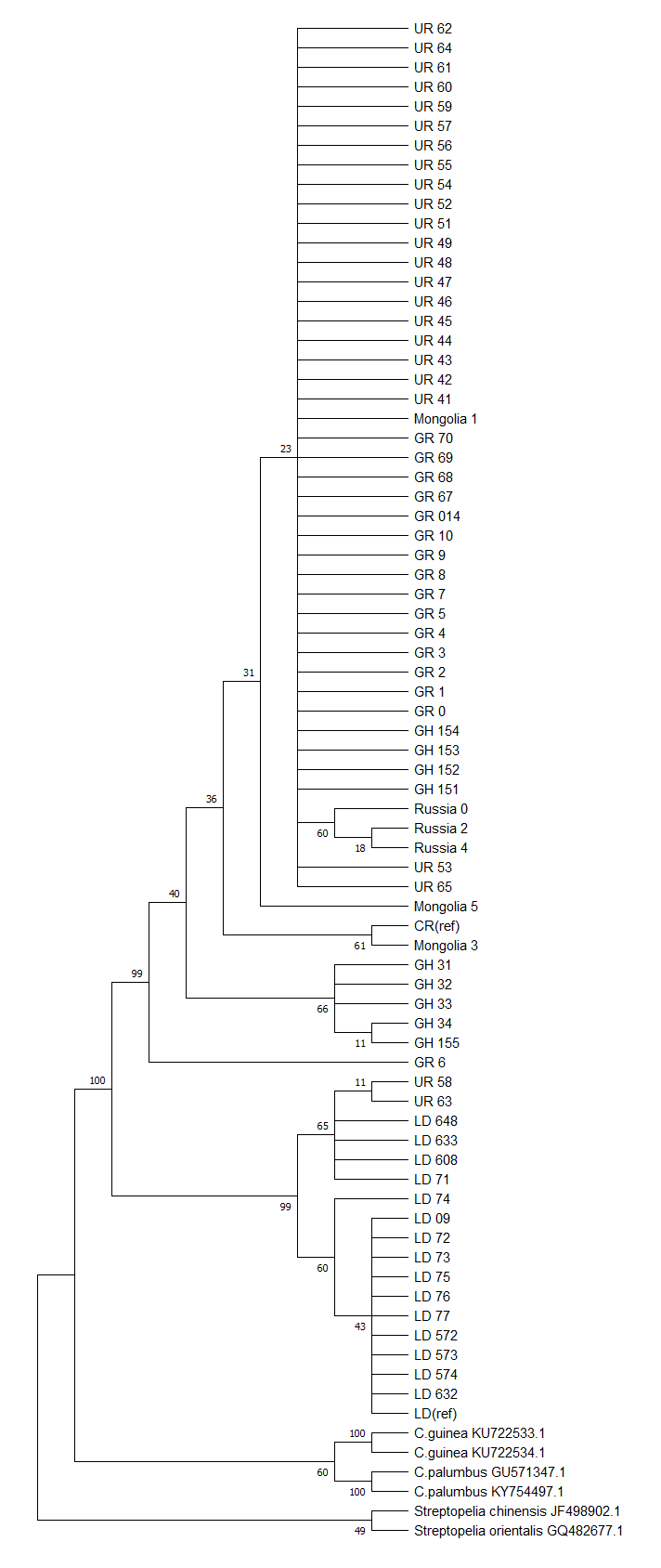

Supplement: Supplementary file 1 — Supplementary file1 (DOCX 46 KB) [file 13258_2021_1212_MOESM1_ESM.docx]
